# Association of smoking cigarettes, age, and sex with serum concentrations of olanzapine in patients with schizophrenia

**DOI:** 10.11613/BM.2023.030702

**Published:** 2023-10-15

**Authors:** Mihovil Horvat, Mate Kadija, Andrijana Ščavničar, Maja Živković, Marina Šagud, Mila Lovrić

**Affiliations:** 1 Faculty of pharmacy and biochemistry, University of Zagreb, Zagreb, Croatia; 2 Department of laboratory diagnostics, University hospital centre Zagreb, Zagreb, Croatia; 3 Clinic for psychiatry, Clinical hospital Vrapče, Zagreb, Croatia; 4 Department of psychiatry and psychological medicine, University hospital centre Zagreb, Zagreb, Croatia; 5 School of medicine, University of Zagreb, Zagreb, Croatia

**Keywords:** central nervous system diseases, olanzapine, smoking, therapeutic drug monitoring

## Abstract

**Introduction:**

Olanzapine is an atypical antipsychotic drug which is effective in the treatment of schizophrenia. Cigarette smoking, age, and sex could be related to the pharmacokinetics and serum concentrations of olanzapine in patients with schizophrenia. The aim of the study was to examine whether there was a significant difference in the serum olanzapine concentrations with regard to the mentioned factors.

**Materials and methods:**

A total of 58 outpatients with schizophrenia (37 smokers, 42 men, 35 older than 40 years) participated in the study. Blood was sampled in serum tubes just before taking the next dose of olanzapine. Olanzapine was extracted by liquid-liquid extraction and was measured by an in-house high-performance liquid chromatography method on Shimadzu Prominence HPLC System with diode array detector SPD-M20A (Shimadzu, Kyoto, Japan). The results were expressed as the ratio of concentration to the daily dose of olanzapine (C/D). Non-parametric statistical tests were used to analyse differences between variables.

**Results:**

The median C/D of olanzapine (interquartile range) in smokers was 6.0 (3.4-10.2) nmol/L/mg and in non-smokers 10.1 (5.9-17.6) nmol/L/mg; P = 0.007. The median C/D of olanzapine in patients younger than 40 years was 5.6 (4.5-10.2) nmol/L/mg and in patients older than 40 years 8.4 (5.6-13.0) nmol/L/mg; P = 0.105. The median C/D of olanzapine in male patients was 6.6 (4.6-10.4) nmol/L/mg and in female patients 9.0 (5.9-15.3) nmol/L/mg; P = 0.064.

**Conclusions:**

The serum olanzapine concentration was significantly lower in smoking than in non-smoking patients with schizophrenia. No significant difference was demonstrated with regard to age and sex.

## Introduction

Olanzapine is an atypical antipsychotic drug used in the treatment of patients with schizophrenia. It is effective in patients with a wide range of psychopathology, including negative symptoms, has a lower frequency of extrapyramidal symptoms, and minimally affects prolactin concentrations (*1*). Olanzapine achieves its pharmacological effect through a combination of dopamine and serotonin type 2 (5HT_2_) receptor antagonism (*2*). However, weight gain and metabolic syndrome with the development of cardiovascular diseases are side effects responsible for shortened life expectancy and poor adherence in patients with schizophrenia (*3*). Although in clinical practice olanzapine (and clozapine) have been associated with the highest risk of metabolic syndrome, their effectiveness is still higher than of other atypical antipsychotics (*3*). For these reasons, olanzapine belongs to the group of antipsychotics (level 1) for which therapeutic drug monitoring (TDM) is strongly recommended (*4*, *5*).

Beside metabolic side effects, an additional problem in optimizing olanzapine dose is smoking given that 70 to 80% of patients with schizophrenia are smokers, with 36% of them smoking 20 cigarettes or more *per* day (*6*, *7*). In clinical practice, dose adjustments may be necessary because smokers may be at risk of subtherapeutic psychopharmacological treatment (*8*). Olanzapine is extensively metabolized by the cytochrome P4501A2 (CYP1A2) enzyme to the inactive product 4’-desmethyl olanzapine (*9*). Smoking can significantly affect the blood concentration of drugs that are CYP1A2 substrates. Polycyclic aromatic hydrocarbons (not nicotine) from cigarette smoke induce the CYP1A2 enzyme through activation of the aryl hydrocarbon receptor (AhR) and increase its activity (*10*). Consequently, this leads to a decrease in the olanzapine blood concentration and ineffective therapy (*5*, *11*). It is assumed that the olanzapine serum concentration also may vary between individuals depending on sex and age. Women are known to have lower CYP1A2 enzyme activity and therefore lower olanzapine clearance than men (*12*). Some studies have shown that effect of age on serum olanzapine concentration becomes more pronounced in older age (*12*). Therapeutic drug monitoring is a valuable tool for personalizing the olanzapine dose in patients with schizophrenia. The correct interpretation of the obtained results and optimization of pharmacotherapy using TDM reduces treatment cost and increases patient safety (*2*).

Because of the known large interindividual variability in olanzapine pharmacokinetics, we hypothesized that age, sex, and lifestyle habits such as cigarette smoking might be associated with serum olanzapine concentrations in patients with schizophrenia. The aim of our study was to examine whether there was a difference in the measured concentrations of olanzapine with regard to the smoking status, sex, and age of patients with schizophrenia.

## Materials and methods

### Subjects

Fifty-eight outpatients who met schizophrenia diagnosing criteria listed in Diagnostic and statistical manual of mental disorders, 4th edition, participated in this study (*13*). Blood was sampled from patients with schizophrenia who needed general biochemical tests. An additional serum tube was sampled to measure olanzapine in the study. All patients were treated and monitored at Department for psychiatry and psychological medicine, University hospital centre (UHC) Zagreb, and Clinic for psychiatry Vrapče in Zagreb, Croatia. The study was conducted in the period from September 2017 to September 2018. The protocol of the study was approved by the Ethics committee of UHC Zagreb on September 19, 2017, in Zagreb (Class: 8.1-17/175-2; Number: 02/21 AG). All patients gave written informed consent; they were in clinically stable remission and were not hospitalized for the previous six months. They were receiving regular maintenance monotherapy in the form of intramuscular long-acting injection of olanzapine pamoate (300 mg or 405 mg *per* month) or oral olanzapine (10 mg, 15 mg, or 20 mg *per* day) available under several different brand names. The patients did not receive additional psychopharmacological therapy for at least three previous months, except for low-dose benzodiazepines (*i.e*., the equivalent of 10 mg diazepam or less) on an as-needed basis. The patients were divided according to the smoking status into groups of non-smokers and smokers, according to sex into male and female group, and according to age into groups of patients younger than 40 years and patients older than 40 years (Table 1). The ratio of male to female patients in the study was 2.6/1. Non-smokers were patients who did not smoke a single cigarette during therapy (0 cigarettes *per* day). The criterion for the patient’s smoking status was at least one cigarette smoked *per* day during olanzapine therapy. Smokers were ranked into four groups according to the number of cigarettes smoked *per* day. Blood was sampled just before taking the next oral dose (24-hour time interval) or just before the next injection (1-month time interval) in patients on the depot formulation of olanzapine. The patients’ blood was sampled in Vacuette tubes 4 mL CAT Serum Clot Activator (Greiner Bio-One, Kremsmünster, Austria). The blood was centrifuged for 5 min at 3500 rpm at room temperature in a centrifuge Rotofix 32 (Hettich Zentrifugen, Tuttlingen, Germany). Serum samples were kept in a refrigerator at + 4°C if the analysis was planned within 24 h. Serum samples that were not analysed within 24 h were stored at - 20°C and were analysed within a week.

**Table 1:** Groups of patients whose blood was analysed in the study with regard to sex, age, route of drug administration, and smoking status

	**Male** **N = 42**	**Female** **N = 16**	**Total** **N = 58**
Age, years	43 (20-64)	45 (29-64)	44 (20-64)
≤ 40 years (N)	19	4	23
> 40 years (N)	23	12	35
i.m. olanzapine (N)	30	8	38
i.m. 405 mg/4 weeks (N)	25	2	27
i.m. 300 mg/4 weeks (N)	5	6	11
p.o. olanzapine (N)	12	8	20
p.o. 20 mg/day (N)	6	3	9
p.o. 15 mg/day (N)	2	2	4
p.o. 10 mg/day (N)	4	3	7
non-smokers (N)	11	10	21
smokers (N)	31	6	37
1-10 cigarettes/day (N)	2	1	3
11-20 cigarettes/day (N)	15	4	19
21-30 cigarettes/day (N)	8	1	9
> 31 cigarettes/day (N)	6	0	6
i.m. - intramuscular. p.o. - *per* oral.			

### Methods

Olanzapine (parent compound) was measured in serum samples by using an in-house method on high-performance liquid chromatography (HPLC) with a diode array detector (DAD). The type of analyser was Shimadzu Prominence HPLC System with SPD-M20A DAD (Shimadzu, Kyoto, Japan). The method was validated according to the Guideline on the validation of bioanalytical methods of the European medicines agency (*14*). Between-day precision, expressed as coefficient of variation (CV), was 8.3% for level 1 (67.3 nmol/L) and 8.4% for level 2 (203.0 nmol/L), which fulfilled the validation criterion of < 10%. To assess the accuracy of the method, the recovery test was used which gave results of 99% for level 1 (67.3 nmol/L) and 101% for level 2 (203.0 nmol/L). The criterion for accuracy was ± 15% (85-115%). The linearity of the method was in the concentration range from 4.8 to 960.0 nmol/L with the correlation coefficient r = 1.00 (criterion r > 0.99). The lower limit of quantification (LLOQ) was 7.8 nmol/L and the limit of detection (LOD) was 2.7 nmol/L. ClinCal Calibrator and Control for psychoactive drugs TDM3 were used for calibration and quality control (Recipe, Munich, Germany). Chloramphenicol (Sigma Aldrich, St. Louis, USA) was used as an internal standard (IS). Before analysing the samples, liquid-liquid extraction was performed. A mixture of ethyl acetate and n-hexane (Merck, Darmstadt, Germany) in a ratio of 1:1 was used as the extraction solvent which evaporated in a water bath at 37°C using a stream of air. The extracted samples were resuspended in a mixture of 10 mM ammonium acetate buffer pH 3.5 (Merck, Darmstadt, Germany), 0.1 M carbonate buffer pH 10.9 (Aldrich Chemical Company Inc., Milwaukee, USA), and mobile phases in a ratio of 1:1:1. Methanol and acetonitrile (Merck, Darmstadt, Germany) were used as mobile phases in a ratio of 1:1 for pump A and ammonium-acetate buffer for pump B. A gradient flow program (1.0 mL/min) of the mobile phase was used for elution from the column and the pressure was 800 psi. Chromatographic separation was carried out on the InertSustainTM C18 column, dimensions 4.6 x 50 mm, 3 μm (Gl Sciences Inc., Tokyo, Japan). The run-time was 20 min, and the injection volume was 50 μL. Detector signal processing and data generation were performed using a computer program EZStart 7.4 (Shimadzu Corporation, Kyoto, Japan). The eluted substances olanzapine and IS were detected at 254 nm. The target therapeutic range for olanzapine, 64-256 nmol/L, was taken from Hiemke *et al.*’s Consensus Guidelines for Therapeutic Drug Monitoring in Neuropsychopharmacology (*4*). Recommended therapeutic concentrations in serum for depot and oral olanzapine formulations are identical because insufficient pharmacokinetic studies have been conducted (*4*). Olanzapine concentration was assessed through external quality assessment schemes Instand External Quality Assessment Schemes for Antipsychotics/Antidepressants (INSTAND e.V., Düsseldorf, Germany).

### Statistical analysis

The distribution of the collected data was evaluated with the D’Agostino-Pearson test. The data did not follow a normal distribution (P < 0.001) and were presented as median and interquartile range. Non-parametric statistical tests were used to analyse differences between variables. The Mann-Whitney test was used to examine differences between groups of patients divided by smoking status, sex, and age. The Kruskal-Wallis test was used to examine differences between groups of patients divided by the number of cigarettes smoked *per* day. The relationship between age and the smoking status of patients was examined using Spearman’s rank correlation coefficient. The proportion of non-smokers and smokers with olanzapine concentrations below the therapeutic range was compared using the chi-square test. A value of P < 0.05 was taken as confirmation of a statistically significant difference. For statistical data analysis, MedCalc v20.110 (MedCalc Software Ltd, Ostend, Belgium) was used. The values of olanzapine serum concentrations (nmol/L) were divided by daily dose of drug (mg) to obtain concentration ratio to the daily dose of olanzapine (C/D) in nmol/L/mg, hereinafter referred to as dose-adjusted concentration. The route influence of drug administration was avoided by using dose-adjusted concentrations. Intramuscular doses of 300 and 405 mg/4 weeks, administered once a month, were converted to daily doses of 10 and 13.5 mg/day. Such uniform values were used in statistical tests.

## Results

**Figure 1:**
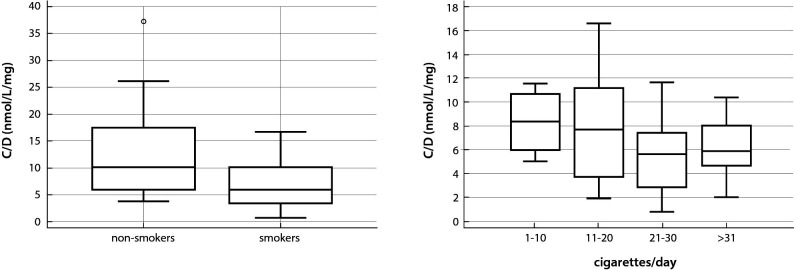
On the left: dose-adjusted concentrations (C/D) of olanzapine in non-smokers and smokers. On the right: dose-adjusted concentrations (C/D) of olanzapine in smokers with regard to the number of cigarettes smoked *per* day. The boxes represent the interquartile range, the middle horizontal line the median, and the vertical line min-max values.

Median trough olanzapine concentrations in both smokers and non-smokers were within therapeutic range for olanzapine, but median trough olanzapine concentrations in smokers were much closer to the lower limit of the therapeutic range (Table 2). Approximately one-third of all patients had the trough olanzapine concentration below the lower limit of the therapeutic range (< 64 nmol/L), and more than two-thirds of them were smokers. Using a chi-square test, it was found that the relation between smoking status and olanzapine serum concentrations below therapeutic range was not significant, P = 0.278. However, median trough and dose-adjusted olanzapine concentrations in smokers were approximately twice as low as in non-smokers (Figure 1). There was a statistically significant difference between dose-adjusted olanzapine concentrations in smokers and non-smokers. Furthermore, there was no statistically significant difference between smokers depending on the number of cigarettes smoked *per* day. On the right box and whisker graph in Figure 1, it is visible that C/D ratios of the first three groups of smokers decrease with an increase in the number of cigarettes smoked, but this decrease is not significant. Furthermore, patients smoking 21-30 cigarettes *per* day and patients smoking more than 30 cigarettes *per* day had approximately similar median C/D of olanzapine (Table 2, Figure 1).

**Table 2:** Results obtained for non-smokers and smokers

	**C** **(nmol/L)**	**C/D** **(nmol/L/mg)**	**P**
Non-smokers (N = 21)	174.3 (65.8-209.1)	10.1 (5.9-17.6)	0.007
Smokers (N = 37)	95.2 (45.9-141.5)	6.0 (3.4-10.2)	
1-10 cigarettes/day (N = 3)	84.2 (72.3-137.3)	8.4 (5.9-10.7)	0.476
11-20 cigarettes/day (N = 19)	102.0 (47.6-150.5)	7.7 (3.8-11.2)	
21-30 cigarettes/day (N = 9)	83.7 (39.5-104.5)	5.6 (2.9-7.4)	
> 31 cigarettes/day (N = 6)	95.2 (63.3-159.0)	5.9 (4.7-8.0)	
Variables are presented as median (interquartile range). Differences between dose-adjusted concentrations of olanzapine in non-smokers and smokers were determined by the Mann-Whitney U test, while differences between smokers with regard to the number of cigarettes smoked were determined by the Kruskal-Wallis test. C - trough concentrations of olanzapine. C/D - dose-adjusted concentrations of olanzapine. P < 0.05 was considered statistically significant.			

**Figure 2:**
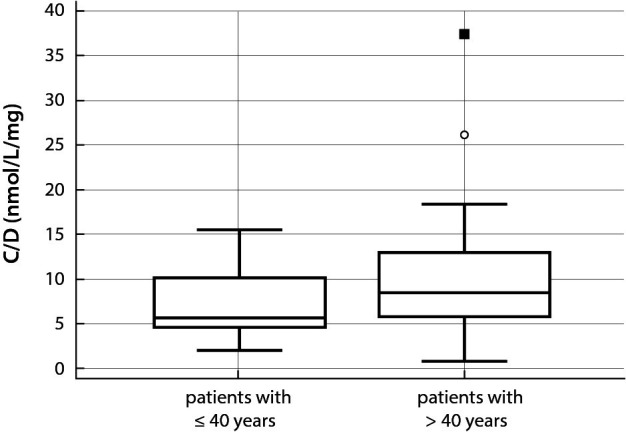
Dose-adjusted concentrations (C/D) of olanzapine in patients with schizophrenia considering the age limit of 40 years. The boxes represent the interquartile range, the middle horizontal line the median, and the vertical line min-max values.

**Figure 3:**
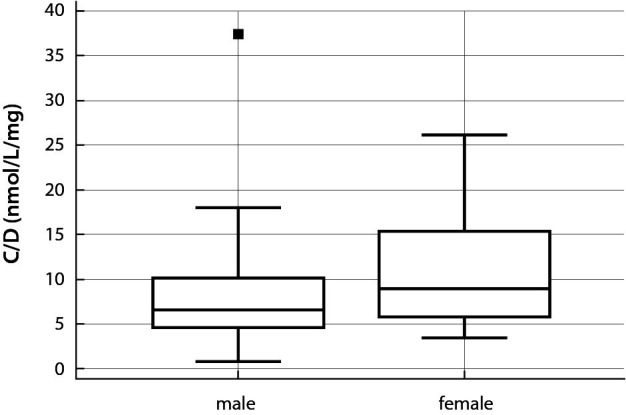
Dose-adjusted concentrations (C/D) of olanzapine in male and female patients with schizophrenia. The boxes represent the interquartile range, the middle horizontal line the median, and the vertical line min-max values.

The influence of age and sex on serum olanzapine concentrations was also examined. The age limit of 40 years was taken as a criterion for dividing patients into two groups by age (Table 3). No difference was found between age groups, *i.e.* between patients younger and older than 40 years regardless of smoking status (Figure 2). Also, no correlation was found between patients’ age and smoking status (Spearman’s coefficient correlation, r_s_ = 0.17; P = 0.204). The difference between dose-adjusted olanzapine concentrations in male and female patients was not statistically significant (Figure 3, Table 4).

**Table 3:** Results obtained considering the age limit of 40 years

	**C** **(nmol/L)**	**C/D** **(nmol/L/mg)**	**P**
≤ 40 years (N = 23)	88.3 (51.7-130.5)	5.6 (4.5-10.2)	0.105
> 40 years (N = 35)	122.5 (61.0-180.6)	8.4 (5.6-13.0)	
Variables are presented as median (interquartile range). Differences in dose-adjusted concentrations of olanzapine were determined by the Mann-Whitney U test. C - trough concentrations of olanzapine. C/D - dose-adjusted concentrations of olanzapine. P < 0.05 was considered statistically significant.			

**Table 4:** Results obtained considering the sex

	**C** **(nmol/L)**	**C/D** **(nmol/L/mg)**	**P**
Male (N = 42)	102.0 (52.1-155.0)	6.6 (4.6-10.4)	0.064
Female (N = 16)	145.0 (59.2-179.0)	9.0 (5.9-15.3)	
Variables are presented as median (interquartile range). Differences in dose-adjusted concentrations of olanzapine were determined by the Mann-Whitney U test. C - trough concentrations of olanzapine. C/D - dose-adjusted concentrations of olanzapine. P < 0.05 was considered statistically significant.			

## Discussion

The results of our study indicate that C/D of olanzapine in smokers is significantly lower than in non-smokers. A higher C/D value indicates a slow metabolism of the drug and its removal, while lower C/D values indicate a rapid removal of the drug from circulation. It is known that smokers have a higher clearance and lower concentrations of olanzapine than non-smokers (*1*). A meta-analysis by Tsuda *et al.* showed that olanzapine doses should be reduced by 30% in non-smokers compared to smokers to obtain equivalent olanzapine concentrations (*15*). A population pharmacokinetic analysis showed that olanzapine clearance may be 37 to 48% higher in smokers (*16*). Furthermore, Heres *et al.* have stated that olanzapine concentrations are higher in non-smokers than in smokers, regardless of olanzapine administration route (*17*). Our results also agree with the results of the above mentioned studies. Further examination showed that the number of cigarettes smoked *per* day did not correlate with reduction in C/D of olanzapine. The meta-analysis by Djordjevic *et al.* found that smoking at least five cigarettes *per* day was associated with reduced efficacy of olanzapine (*11*). Haslemo *et al.* have concluded that daily consumption of 7-12 cigarettes is probably sufficient for maximum induction of olanzapine metabolism (*18*). Moschny *et al.* have stated that maximum induction of CYP1A2 is achieved after smoking 10 cigarettes *per* day and that smoking 10 or 30 cigarettes *per* day does not cause a difference in serum concentrations of olanzapine (*10*). Additionally, Carrillo *et al.* found that non-smokers and those who smoke four or less cigarettes *per* day had similar CYP1A2 activity (*9*). The consensus guidelines published by Hiemke *et al.* state that the effects of smoking should be taken into account when smoking more than 10 cigarettes *per* day (*4*). Serum olanzapine concentrations below therapeutic range are not related to the patients’ smoking status. Our finding is consistent with the fact that there are no guidelines in the literature for adjusting the olanzapine dose according to smoking status.

The median C/D of olanzapine in patients older than 40 years is not significantly higher than in patients younger than 40 years. It is assumed that concentration of olanzapine will be higher the older the patient is (*12*). In this study, an arbitrary cut-off value of 40 years was taken, so older patients were considered to be those over 40 years old. The oldest patient was 64 years old, so the group of elderly patients was heterogeneous. A study by Castberg *et al.* which included more than 3000 patients (the oldest patient was 86 years old) showed that every five years after the age of 40, dose-adjusted concentration of olanzapine increased by 1.7% in men and 1.3% in women (*12*). Another study has found an increase of about 27% in dose-adjusted olanzapine concentrations in patients over 60 years of age compared with younger subjects. It should be noted that studies examining the effect of age on olanzapine concentrations are limited due to the small number of very old patients, over 85 years of age (*12*). This was also the case in our study. Some authors state that olanzapine prescription rates decrease slightly in elderly patients, and if olanzapine is prescribed, a lower starting dose (5 mg) is recommended (*19*). In elderly people, the activity of the CYP1A2 enzyme remains preserved but organ perfusion (liver and kidney) and circulation weaken so that metabolism and elimination of olanzapine can be slowed down (*4*). The most likely mechanism underlying the higher dose-adjusted concentrations in the elderly is a lowered hepatic clearance of the drugs caused by a decreased intrinsic hepatic metabolic function, lower liver volume, and/or reduced hepatic blood flow due to, for example, impaired cardiac output (*12*). The drug metabolism may also be altered due to kidney and liver diseases.

In our study, no statistically significant difference was obtained in dose-adjusted olanzapine concentrations according to sex. In an extensive study based on the results from the routine TDM database (16,171 samples for olanzapine), it was shown that dose-adjusted olanzapine concentrations in women were 26.1% higher than in men (*12*). The reason for the higher olanzapine concentration-dose ratio in women may be that CYP1A2 also metabolizes some female sex hormones (*20*). Therefore, there is competition between CYP1A2 substrate drugs and hormones. This observation is most pronounced in late pregnancy (*20*). In our study, there were no data related to the pregnancy of patients. Another fact is that olanzapine absorption is higher in women than in men due to slower gastric emptying and a longer gastrointestinal transit time (*21*). In addition, the slower elimination and higher volume of olanzapine distribution in women lead to a longer half-life of olanzapine and slower clearance (*21*). Other possible mechanisms include sex differences in blood flow and liver size, as well as differences in the expression of metabolizing enzymes and transporters (*22*). According to Eli Lilly and Company, no apparent differences between men and women have been demonstrated in the efficacy or adverse effects of the drug although olanzapine clearance is approximately 30% lower in women than in men (*23*). Although olanzapine efficacy and safety depend on dose, sex, age and CYP1A2 genotype, cigarette smoking remains the most important factor affecting olanzapine therapy (*11*). Similarly, Moschny *et al.* concluded that among factors such as age, sex, origin, and co-medication, smoking behaviour had the greatest effect on olanzapine concentrations (*10*).

A main limitation of the study is the small number of samples, especially in subgroups after ranking. Although the cumulative effect of smoking, age, and sex can lead to significant population pharmacokinetic differences, it was not examined due to the insufficient number of participants in our study (*23*). There was also an uneven number of patients within certain groups. Most of the participants in the study were men, most of whom were smokers, while the women were mostly non-smokers. The presence of gene polymorphisms among patients was not examined. Data on other comorbidities of the patients who participated in the study were not collected.

In conclusion, this study proved that smoking is associated with lower serum olanzapine concentrations. No significant differences were found in olanzapine serum concentrations with regard to age and sex in patients with schizophrenia.
